# The role of gonadal hormones and sex chromosomes in sex-dependent effects of early nutrition on metabolic health

**DOI:** 10.3389/fendo.2023.1304050

**Published:** 2023-12-22

**Authors:** Julian K. Christians, Karen Reue

**Affiliations:** ^1^ Department of Biological Sciences, Simon Fraser University, Burnaby, BC, Canada; ^2^ Centre for Cell Biology, Development and Disease, Simon Fraser University, Burnaby, BC, Canada; ^3^ British Columbia Children’s Hospital Research Institute, Vancouver, BC, Canada; ^4^ Women’s Health Research Institute, BC Women’s Hospital and Health Centre, Vancouver, BC, Canada; ^5^ Department of Human Genetics, David Geffen School of Medicine, University of California, Los Angeles, Los Angeles, CA, United States

**Keywords:** sex chromosomes, gonadal sex, Four Core Genotypes, gonadectomy, developmental origins of health and disease, fetal programming, genome-wide association studies, quantitative trait loci

## Abstract

Early-life conditions such as prenatal nutrition can have long-term effects on metabolic health, and these effects may differ between males and females. Understanding the biological mechanisms underlying sex differences in the response to early-life environment will improve interventions, but few such mechanisms have been identified, and there is no overall framework for understanding sex differences. Biological sex differences may be due to chromosomal sex, gonadal sex, or interactions between the two. This review describes approaches to distinguish between the roles of chromosomal and gonadal sex, and summarizes findings regarding sex differences in metabolism. The Four Core Genotypes (FCG) mouse model allows dissociation of the sex chromosome genotype from gonadal type, whereas the XY* mouse model can be used to distinguish effects of X chromosome dosage vs the presence of the Y chromosome. Gonadectomy can be used to distinguish between organizational (permanent) and activational (reversible) effects of sex hormones. Baseline sex differences in a variety of metabolic traits are influenced by both activational and organizational effects of gonadal hormones, as well as sex chromosome complement. Thus far, these approaches have not been widely applied to examine sex-dependent effects of prenatal conditions, although a number of studies have found activational effects of estradiol to be protective against the development of hypertension following early-life adversity. Genes that escape X chromosome inactivation (XCI), such as *Kdm5c*, contribute to baseline sex-differences in metabolism, while *Ogt*, another XCI escapee, leads to sex-dependent responses to prenatal maternal stress. Genome-wide approaches to the study of sex differences include mapping genetic loci influencing metabolic traits in a sex-dependent manner. Seeking enrichment for binding sites of hormone receptors among genes showing sexually-dimorphic expression can elucidate the relative roles of hormones. Using the approaches described herein to identify mechanisms underlying sex-dependent effects of early nutrition on metabolic health may enable the identification of fundamental mechanisms and potential interventions.

## Introduction

Early-life conditions such as prenatal nutrition can have long-term effects on metabolic health, and these effects may differ between males and females (1–6). Moreover, the effectiveness of potential interventions may depend on sex (7). Understanding the biological mechanisms underlying such sex-dependent responses will improve interventions by, for example, revealing why one sex is protected or the other sex is vulnerable. However, few mechanisms underlying sex differences in the response to early-life environment have been identified, and there is no established framework for investigating sex differences, or for predicting sex-dependence. In contrast, there has been substantial work on the mechanisms underlying baseline sex differences in metabolism (8–16), i.e., differences between the sexes in the general population and in animal models not manipulated in early life. The application of similar powerful approaches to understand sex-dependent developmental programming would be valuable.

Biological sex differences can be ascribed to two factors: genetic sex, which is determined at conception by inheritance of XX or XY chromosomes, and gonadal sex, which is specified by the sex chromosomes and influences the hormonal milieu at specific times during development and in adulthood. In many cases, there are interactions between chromosomal and gonadal sex to influence a particular trait. Additionally, there are interactions between biological sex and gender, which relates to cultural and behavioral norms of femininity and masculinity. The intersection of biological sex and gender likely contributes to sex differences in the prevalence and presentation of numerous diseases. However, in this review, we will use the term “sex” for brevity because we are primarily concerned with exposures that occur prior to birth and will often refer to animal models.

Most work on sex differences in metabolism has focused on the role of gonadal hormones, or simply assumes that sex differences in adulthood are due to hormonal effects. However, the emergence of sex differences (e.g., in gene expression) prior to the development of gonads (17, 18) illustrates the importance of chromosomal sex, but what physiological processes are affected, and how this influences interactions between the early environment and health later in life are not clear. The roles of the sex chromosomes can be further categorized into the role of X chromosome dosage vs the presence of the Y chromosome. Similarly, the role of sex hormones can be divided into organizational (permanent) and activational (reversible) effects (19).

The purpose of this review is to provide an overview of approaches that allow the discrimination of specific sex components that explain baseline sex differences in metabolism, to describe how they have been applied, and to propose how they might be valuable in studies of developmental programming. In particular, we focus on strategies to uncover the relative importance of gonadal hormones and sex chromosomes in the development of sex-biased effects of early nutrition on metabolic health.

## Approaches to dissect the roles of sex chromosomes and hormones

**Figure 1:**
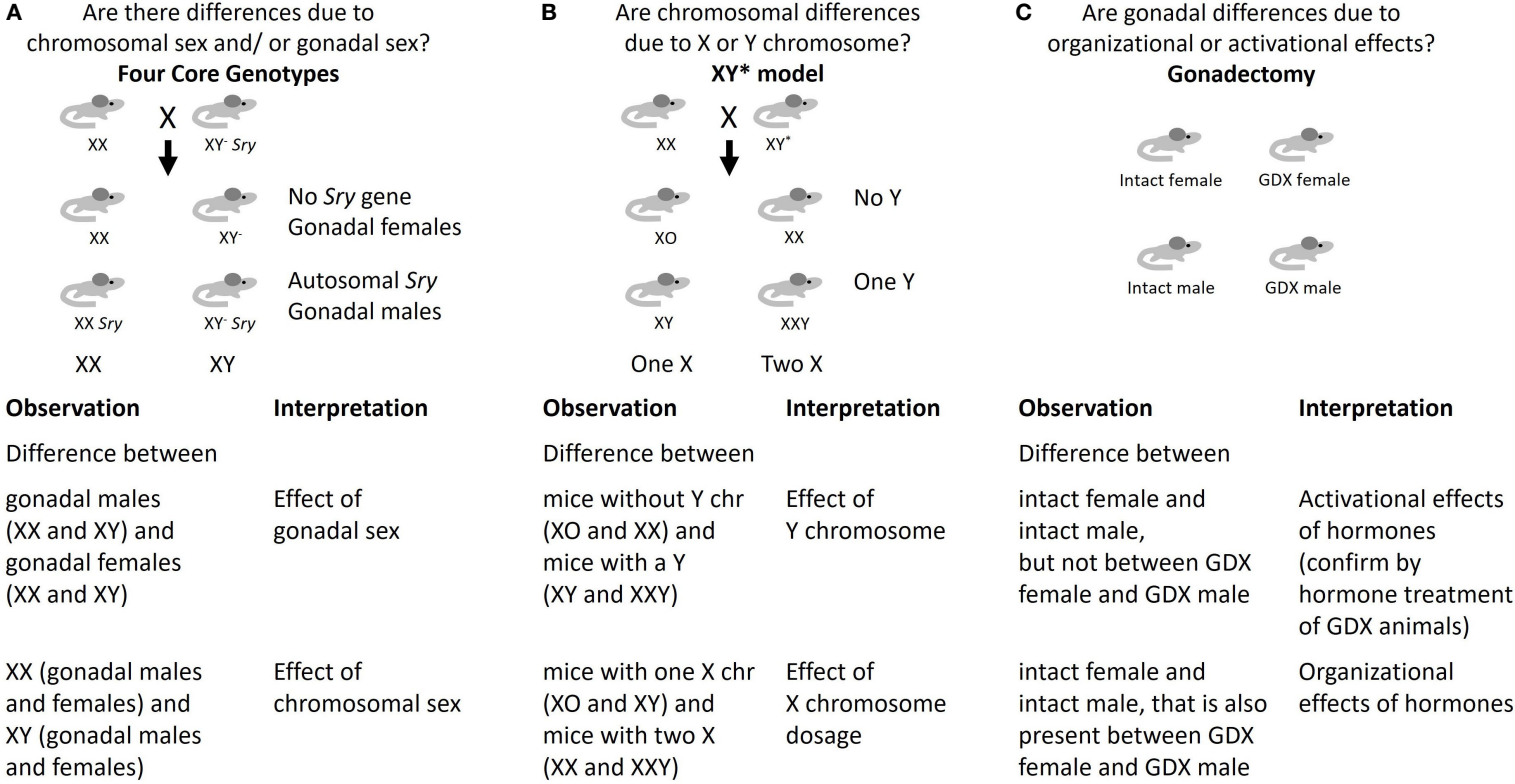
Approaches to identify mechanisms underlying sex differences. **(A)** Four Core Genotypes mouse model; **(B)** XY* mouse model; **(C)** Gonadectomy.

Using standard experimental models, it has not been possible to distinguish potential effects of sex chromosomes and gonadal hormones on responses to early life conditions. Although not yet used to study developmental programming, the Four Core Genotypes (FCG) mouse model is the most widely-used system for examining the relative roles of gonadal and chromosomal sex (20, 21). This model allows dissociation of the sex chromosome genotype (XX or XY) from gonadal type (ovaries or testes) so that the impact of each on traits of interest may be evaluated. The model combines a deletion in the testis-determining *Sry* gene from the Y chromosome (referred to as Y^-^), with an insertion of the *Sry* gene on an autosome (12, 16), such that the determination of gonadal sex is independent from the sex chromosomes. As shown in **Figure 1A**, mating between an XY^-^ *Sry* male with a wild-type XX female yields 4 genotypes: XX *Sry* males (with testes), XY^-^ females (with ovaries), as well as normal XX females and XY^-^ *Sry* males (carrying *Sry* on an autosome rather than the Y chromosome). Analysis of a trait of interest using the FCG model reveals whether a trait is influenced by chromosomal sex, gonadal sex, or an interaction between the two. Depending on the results, subsequent analyses may involve further investigation of the sex chromosome effect or gonadal effect as described below.

If analysis with FCG mice shows that XX mice (including both gonadal females and gonadal males) differ from XY^-^ mice (including both gonadal females and gonadal males), this indicates that chromosomal sex is a determinant. In that case, it becomes valuable to determine whether dosage of the X or Y chromosome is causal. This can be approached with the XY* mouse model, which allows the generation of mice with XO, XX, XY or XXY chromosome complements due to the ability of the Y* chromosome to undergo abnormal recombination events (12). Analysis of the trait of interest in XY* mice reveals whether it is influenced by presence of one or two X chromosomes, or presence or absence of a Y chromosome (**Figure 1B**). This can aid in the identification of potential candidate genes on the X or Y chromosome. Studies in humans with the same sex chromosome genotypes as in the XY* model, such as XO (Turner syndrome) and XXY (Klinefelter syndrome), have corroborated sex chromosome effects observed in XY* mice in traits such as adiposity and development of metabolic syndrome (15). However, studies of these genotypes in humans are challenging due to limited numbers of subjects and complications due to exogenous hormone treatment.

If analysis with FCG mice indicates that a trait segregates with *Sry* genotype (i.e., mice with testes differ from mice with ovaries), it is attributed to gonadal sex (**Figure 1A**). In this case, gonadectomy in adult mice can be used to further distinguish organizational and activational effects of gonadal hormones (**Figure 1C**) (16). While gonadectomy is useful for identifying activational effects of the sex hormones even in standard male and female mice, organizational effects of sex hormones are confounded with the effects of chromosomal sex unless the FCG model is used. Deeper investigation of gonadal sex effects may also be performed by gonadectomy followed by replacement of specific gonadal hormones, and by ablation of estrogen and androgen hormone action with selective agonists or by genetic deletion (21–23).

## Results from mouse models that dissect the roles of sex chromosomes and hormones

Studies in FCG and XY* mouse models have identified specific roles for chromosomal and gonadal sex in numerous physiological traits including behavior, neurological diseases, atherosclerosis, and obesity (12, 24–30). These studies are aided by the availability of FCG and XY* strains with a C57BL/6 inbred genetic background, which is susceptible to numerous neurologic, immunologic and metabolic diseases. The earliest studies of metabolism in FCG mice assessed the determinants of sex differences in body weight, which showed that this trait is influenced by both activational and organizational effects of gonadal hormones, as well as sex chromosome complement. As is typical in mouse models, body weight was greater in gonadally intact males than females, but XX mice were heavier than XY mice (28, 31). XX chromosome complement was also associated with increased adipose tissue mass, fatty liver, higher plasma cholesterol levels, and development of atherosclerotic lesions in the aortic sinus (25, 28, 29, 31). Gonadal sex, particularly via the activational effects of sex hormones, was the predominant determinant of circulating triglyceride levels, and had major effects on gene expression in liver, adipose tissue, and hippocampus of FCG mice, although sex chromosomes influenced expression of specific genes as well (29, 32–35). There were also interactions between gonadal sex and chromosomal sex, for example, in fat and lean body composition (28, 30, 31).

One component of energy balance that contributes to sex differences in adiposity is food consumption. Interestingly, the effects of gonadal hormones and sex chromosomes on food consumption differed between the dark and light phases, with X chromosome copy number influencing food intake specifically during the inactive phase of the circadian cycle (28, 31). Gonadectomized XY mice consumed more of a palatable food and were more motivated to obtain it than XX mice (36). Additional work showed that both chromosome complement and activational effects of gonadal hormones influenced circadian regulation, particularly in males (37).

Studies in inbred C57BL/6 XY* mice revealed that differences in adiposity that are associated with chromosomal sex were due to number of X chromosomes, rather than presence of the Y (28). However, on an outbred MF1 genetic background, the presence of a Y chromosome, even in the absence of testes, could influence adiposity, indicating a role for non-*Sry* Y chromosome genes, perhaps including Y-linked genes that are paralogous to X-linked genes (38). In some studies, removing the activational effects of gonadal hormones by gonadectomy uncovered sex differences that were not apparent in intact mice, suggesting that some activational effects of hormones may counterbalance chromosomal effects, causing intact XX females and XY males to be more similar to one another (29). Gonadal and chromosomal sex influencing a given trait in opposite directions appears to be a general phenomenon beyond metabolic traits (27, 39).

To date, no study has used the Four Core Genotypes model to examine sex-dependent effects of prenatal nutrition. However, a few studies have examined interactions between postnatal nutrition, gonadal hormones and sex chromosomes on metabolic traits. The effects of gonadal and chromosomal sex on adiposity are often similar on regular and high-fat diets (28), although an XX complement exacerbated the effects of a high-fat diet on adiposity (30), and XX mice also developed fatty liver and reduced insulin sensitivity (28). In some cases, the relative roles of gonadal and chromosomal sex are affected by diet. Whereas adipose tissue miRNAs were affected primarily by the activational effect of gonadal hormones on a regular diet, a high-fat diet revealed organizational and chromosomal effects (40). Similarly, the effects of chromosomes, gonadal sex, and gonadectomy on plasma lipid traits varied between regular and high cholesterol diets (29). In a model of hypercholesterolemia brought on by genetic deletion of apolipoprotein E, gonadal but not chromosomal sex had a significant effect on total plasma cholesterol and free fatty acids, with higher levels in gonadal males (35), as also occurs in wildtype mice (29). In this model of hypercholesterolemia, gonadal sex and chromosomal sex both widely influenced hepatic gene expression, with more than 3000 genes showing differential expression due to ovaries vs. testes, and ~1400 genes having differential expression in XX vs. XY mice (35). Importantly, differentially expressed genes influenced by gonadal and chromosomal sex were similarly distributed across autosomes and the X chromosome (35).

## Gonadectomy to study sex-dependent effects of early life environment

Gonadectomy has been widely used to investigate the role of acute gonadal hormone action in sex differences, particularly in rodents. However, relatively few studies have used this approach to investigate sex-dependence of developmental programming, although a series of studies have examined activational effects of hormones on the development of hypertension following intrauterine growth restriction in rats (41). Intrauterine growth restriction, induced by surgically reduced uteroplacental perfusion, leads to hypertension in males but not females. Castration in males eliminated the hypertensive response to growth restriction (42). In contrast, ovariectomy revealed susceptibility to growth-restriction induced hypertension, which was rescued by exogenous estradiol (43). These results indicate activational, permissive effects of testosterone and protective effects of estradiol. Further studies have used gonadectomy to further examine the mechanisms underlying these effects, such as renal sensitivity to angiotensin II (44–47).

A maternal low-protein diet also increases blood pressure in rats, sometimes in both sexes (48), and sometimes in males only (49). In contrast to the effects of castration on growth-restriction induced hypertension, castration did not eliminate maternal low-protein diet-induced hypertension (49). At younger ages (16 weeks of age), ovariectomy exacerbated the effects of a maternal low-protein diet on blood pressure, which was partially reversed by estradiol, again suggesting protective activational effects of estradiol (50). However, at 12 months of age, ovariectomy ameliorated effects of a maternal low-protein diet on blood pressure (51). While ovariectomy also increased urinary albumin and protein and urine volume, these effects were independent of maternal diet, indicating that gonadal status did not influence the response to early-life nutrition (51).

Perinatal nicotine exposure increases angiotensin II-induced hypertension in males but not females (52), and ovariectomy increased the response in nicotine-exposed females, which was rescued by exogenous estradiol (53), suggesting protective activational effects of estradiol. Maternal separation, a model of early life stress, did not increase baseline blood pressure, but enhanced angiotensin II-induced hypertension in male rats, and this effect was attenuated by castration (54), consistent with a permissive activational effect of testosterone. While these studies generally suggest that the sex-dependent programming of hypertension is due to activational effects of sex steroids, the FCG model has also shown effects of chromosomal complement on angiotensin II-induced hypertension in the absence of prenatal insults (55).

## Genes that escape X chromosome inactivation

In mammals, X chromosome gene dosage is partially normalized between XX and XY cells by transcriptional inactivation of most genes on one X chromosome in females during early development. However, specific X-linked genes escape X-chromosome inactivation (XCI), sometimes in a tissue-specific manner (56–59). Genes that escape XCI are good candidates for the causative agents responsible for effects of sex chromosomes. Moreover, some of these genes are conserved between rodents and humans (60), such that their study in the former could inform sex differences in the latter. In the studies of sex differences in adiposity described above, a number of XCI escapees, including *Kdm5c*, had higher expression in XX liver and adipose tissue than in the corresponding tissue of XY mice (21, 23). *Kdm5c* encodes a histone demethylase that modifies histone marks at gene promoters and enhancers to influence gene expression across the genome. Hemizygous *Kdm5c* knockout XX mice, with one functional allele and one knockout allele (i.e., gene dosage similar to XY mice) showed that *Kdm5c* dosage influences adiposity and that modifying *Kdm5c* gene dosage mirrors many differences in metabolism that occur between XX and XY mice (30). The human *KDM5C* gene also escapes XCI and *KDM5C* expression levels and genetic variants are both associated with body mass (30).

The X-linked gene O-linked N-acetylglucosamine transferase (*Ogt*) gene undergoes random XCI in most adult tissues (61, 62) but escapes XCI in extraembryonic tissues. As a result, expression is higher in female placentas than in male placentas in both mice and humans (63–65). As with *Kdm5c*, the protein encoded by *Ogt* plays a role in histone modification, such that its sexual dimorphism could have cascading effects on many other genes (66). In the mouse, placental expression of *Ogt* exerts effects on fetal development, influencing fetal hypothalamic gene expression (66), leading to sex-dependent responses to prenatal maternal stress (64, 67). As with *Kdm5c*, the role of *Ogt* in sex differences has been investigated by hemizygous deletion to render placental dosage similar in males and females (66). While *Ogt* would be expected to be responsive to nutrition (68, 69), it is not clear whether placental OGT mediates sex-dependent responses to maternal nutrition. Placental *Ogt* expression was upregulated by a high-fat diet and a low-fat diet (both compared to an intermediate control) in one study (70), but was not affected by other obesogenic diets (71, 72) or a low protein diet (73). The placental expression of *Kdm5c* was not affected by any of these diets.

Other genes escaping XCI, such as spermine synthase (SMS), have widespread effects on placental metabolism and gene expression (74) and so might have sex-dependent effects on the placental response to prenatal nutrition. More generally, genes escaping XCI have higher expression in females, and thus any insult or environmental perturbation that affects the expression of such a gene would be expected to have a proportionately larger effect on gene expression in males. This leads to the prediction that, where sex differences are due to escape from XCI, males should be more affected by early-life environment, as is the case with the effects of prenatal stress mediated by *Ogt* (64, 67).

While specific genes that escape X chromosome inactivation have been examined, the role of Y chromosome genes is less clear. As described above, in some genetic backgrounds the presence of a Y chromosome can influence adiposity (38). Moreover, interactions between the X and Y chromosomes may influence placental weight (75). While these effects on placental weight were observed in the context of interspecific hybrids, they may also occur in placental responses to the environment. Beyond the placenta, some Y chromosome genes are broadly expressed in adult tissues and may balance the expression of X-linked genes that escape XCI (76). These include regulatory genes and so could influence the expression of autosomal genes, leading to lasting effects on the offspring (76).

## Genome-wide approaches

Beyond candidate genes on the sex chromosomes, genetic mapping across the genome has identified autosomal loci that influence traits in a sex-biased manner. Quantitative trait locus (QTL) and genome-wide association studies (GWAS) both seek to identify associations between variation at the DNA level (e.g., single-nucleotide polymorphisms, SNP) and phenotypic variation. In humans, GWAS studies have identified autosomal loci affecting traits related to fat distribution in a sex-biased manner (11, 16, 77, 78). GWAS studies identify associations between genomic regions and phenotypic traits and not the underlying mechanisms or even, in most cases, the underlying genes. However, there are diverse potential mechanisms by which an autosomal locus could have sex-dependent effects, e.g., affecting the expression of sex-steroid receptors or sex-steroid synthesizing enzymes, interacting with proteins coded by Y-chromosome genes or genes that escape XCI, or acting as transcription factors that affect expression of X- or Y-chromosome genes. GWAS is a powerful approach, but its power has not been fully realized for the analysis of genetic determinants of sex differences because many studies fail to segregate results by sex. Additionally, GWAS studies often exclude the sex chromosomes since they require more complex statistical methods in the analysis (16), an issue also encountered in methylation studies (79).

Mouse models are also valuable for genetic mapping of loci affecting traits in a sex-dependent manner. Genome-wide analyses in mice have identified sex-specific loci affecting insulin resistance, adiposity, plasma triglycerides and liver triglycerides (80, 81). Various populations are available for mapping QTL in mice (82), including the Hybrid Mouse Diversity Panel. This panel of >100 mouse strains (83) has been phenotyped for numerous metabolic traits (81, 84) and genotyped at markers throughout the genome (85), allowing association of phenotype and genotype data to identify associated loci. In principle, it would be feasible to acquire a set of strains, examine effects of early life nutrition on metabolic traits, and map loci for the interactions between sex and prenatal environment. However, using such approaches to identify loci influencing interactions between sex and prenatal or postnatal diet will be challenging. Resources are typically designed to detect associations between a trait and genotype, whereas identifying an interaction in the effects of genotype and sex on a trait requires greater statistical power, and identifying a three-way interaction between genotype, sex and prenatal or postnatal diet will pose an even greater challenge. Genome-wide genetic approaches also rely on genetic variation; they may identify loci underlying differences between individuals, but not necessarily those that are involved in responses in all individuals of a given sex where there is no genetic variation. Nevertheless, such approaches could reveal pathways that influence the impact of sex on pre- and postnatal nutrition that are generalizable across different genetic backgrounds.

In addition to identifying associations between genetic variation and metabolic traits, many studies examine associations with gene expression levels to identify expression QTL (eQTL). This allows colocalization between loci that influence gene expression and loci that influence metabolic traits (86), which can contribute to identifying causal genes underlying sex differences (81). eQTL are often sex-dependent in tissue-specific ways (81, 86, 87). However, mapping of eQTL to identify roles of sex chromosomes, i.e., by mapping eQTL to sex chromosomes, would be challenging. Most eQTL studies focus on associations of variants close to a gene (such as in a gene promoter) with the expression of that gene, identifying what are known as cis eQTL. To test associations of variants on the sex chromosomes with expression of genes throughout the genome, which would identify trans-acting eQTL, would require a larger search space, and consequently a larger number of comparisons and reduced statistical power.

Genome-wide genetic approaches can also provide insight into the role of gonadal hormones on gene expression by detecting colocalization between eQTL influencing genes with sex differences in expression and regulatory elements with estrogen receptor or androgen receptor binding motifs (88). The enrichment of known transcription factor binding sites in close proximity to genes with sexually dimorphic expression can also be assessed independently of eQTL. Genes expressed in a sexually dimorphic manner often exhibit enrichment for androgen or estrogen receptor binding sites, consistent with activational effects of hormones playing a prominent role in gene expression (32, 33), although this is not always the case (87). In fact, binding sites for transcription factors other than estrogen and androgen receptors show the greatest enrichment at genes with sexually dimorphic expression (86). For example, binding sites for NR3C1, the glucocorticoid receptor, are sometimes more enriched than binding sites for androgen and estrogen receptors at genes with differential expression regulated by activational effects of sex steroids in mouse liver and adipose tissue (33). Thus, while activational effects of sex steroids contribute to sex differences in gene expression, other transcription factors are also important.

Further study is required to understand the intricate interactions between gonadal hormones and transcription factors that influence sex-biased gene expression. The construction of co-expression networks in combination with transcription factor binding site data has shown that sex steroid receptors are not the predominant transcription factors controlling expression networks, but that these receptors target different genes in males and females in tissue-specific ways (89). This provides additional evidence that transcription factors other than gonadal hormone receptors play an important role in regulating sex-biased gene expression. Applying such analyses to studies of the effects of early nutrition (e.g (70–73).,) could contribute to understanding critical transcription factors and regulatory networks.

## Limitations, gaps and future directions

Identifying mechanisms underlying sex-dependent effects of early nutrition on metabolic health requires consistent sex-dependent effects. Unfortunately, results are often not consistent between studies; in a review of the sex-dependent effects of impaired prenatal nutrition in rodents, the only consistent effect on long-term health was that males were more susceptible to hypertension (90), and similar inconsistencies have been observed in human studies (91). A contributing factor to these inconsistencies is that many studies rely on inadequate statistical approaches, such as analyzing males and females separately and interpreting effects to be sex-dependent when they are significant in one sex but not the other. However, analyzing the sexes separately, without explicitly testing whether the effect in males differs from the effect in females using rigorous approaches (e.g., testing for statistical interactions), will increase the rate of false positives (92, 93). Beyond inconsistencies due to spurious results, there may be real differences between studies where effects of sex on metabolic traits depend on genetic background (81). Reflecting the literature in this field, this review has focused largely on rodents, but studies in rodents may not always recapitulate mechanisms that operate in humans. One difference between species is that more genes escape XCI in humans than in mice, but approximately half of those genes that escape in mice also escape in humans (60), making mice a valid model to study that subset of genes and suggesting that the impact of XCI genes may be even more pronounced in human biology. Furthermore, identifying what is conserved and what has diverged will improve understanding of species differences, which can identify questions of interest to be addressed in humans (12).

We have provided an overview of approaches that are widely used to study sex differences yet remain underutilized in the field of fetal programming. Key approaches include the use of gonadectomy to study activational effects of gonadal hormones, and FCG and XY* mouse models to reveal relative effects of gonadal and chromosomal sex components. The FCG model has been used to study sex differences in a maternal antibody-induced model of autism spectrum disorder (94) and could be applied in an analogous manner to other developmental programming models. Given the known roles of *Ogt* and *Kdm5c* (discussed above), genes that escape XCI deserve further investigation for their roles in sex differences. The placenta likely plays an important role in sex-dependent programming (1, 3, 95, 96), and the FCG model could be used to study sex differences in this tissue given that it is the same genotype as the fetus. In humans in early pregnancy, the production of fetal androgens does not increase differences in placental gene expression between males and females (97), suggesting important roles of sex chromosomes in this tissue.

Identifying the roles of chromosomes and hormones in sex-dependent effects of early nutrition on metabolic health may allow us to move beyond simply cataloging sex differences and to identify general patterns, such as whether sex differences due to hormones or chromosomes are more responsive to prenatal insults. Understanding these mechanisms could stimulate further areas of research, including identification of interactions between glucocorticoids and sex steroids in metabolic programming (98), and sex-dependent responses to pharmaceutical intervention (35).

## Author contributions

JC: Writing – original draft, Writing – review & editing. KR: Writing – review & editing.
